# Efficacy and safety of endoscopic stent placement for afferent loop obstruction using a short double‐balloon endoscopy

**DOI:** 10.1002/deo2.154

**Published:** 2022-07-22

**Authors:** Takashi Ito, Masaaki Shimatani, Masataka Masuda, Koh Nakamaru, Toshiyuki Mitsuyama, Norimasa Fukata, Tsukasa Ikeura, Makoto Takaoka, Kazuichi Okazaki, Makoto Naganuma

**Affiliations:** ^1^ The Third Department of Internal Medicine Division of Gastroenterology and Hepatology Kansai Medical University Osaka Japan; ^2^ Division of Gastroenterology and Hepatology Kansai Medical University Medical Center Osaka Japan; ^3^ Department of Internal Medicine Kansai Medical University Kori Hospital Osaka Osaka Japan

**Keywords:** afferent loop obstruction, benign and malignant, double‐balloon endoscopy, efficacy and safety, endoscopic stent placement

## Abstract

**Background:**

Afferent loop obstruction (ALO) is a rare mechanical complication that occurs after gastrojejunostomy. Recently the use of double‐balloon endoscopy (DBE) can be useful for benign and malignant ALO.

**Methods:**

We retrospectively identified 22 patients who underwent DBE for ALO from January 2009 to December 2020. We evaluated the efficacy and safety of short DBE treatment for ALO.

**Results:**

Short DBE was able to reach an obstructive site in the afferent limb in all patients (100%) and was able to reach the blind end in 14 patients (64%). The technical success rate was 100%, and the clinical success rate was 95%. Procedure‐related adverse events occurred in two patients (9%). In the case of benign ALO, three of nine patients showed improvement in ALO with single stent placement. Two of nine patients improved after the replacement of the plastic stent (PS) two or three times. Four of nine patients continued with the replacement of PS. In the case of malignant ALO, the metallic stent was placed in 10 patients, and eight patients with metallic stents did not experience stent occlusion until they died. Reintervention was attempted in six patients (27%) with benign ALO but was not attempted in malignant ALO.

**Conclusions:**

Treatments for ALO using the short DBE was effective and relatively safe because the technical and clinical success rates were very high and there were relatively low complications. Consequently, short DBE could be the first‐choice treatment for both benign and malignant ALO.

## INTRODUCTION

Afferent loop obstruction (ALO) is a rare mechanical complication that occurs after gastrojejunostomy. In most cases, ALO may develop in association with Billroth II (B‐II) or Roux‐en‐Y (R‐Y) gastrectomy or pancreaticoduodenectomy (PD).^1^ ALO has been reported in 0.3%–1.0% of patients after gastrectomy.^2^, ^3^ ALO causes an increase in the internal pressure, which leads to distension along with the accumulation of biliary, pancreatic, and enteric secretions. Subsequently, it often leads to the development of vomiting, jaundice, or cholangitis. Various etiologies of ALO have been reported, and the treatment differs between benign and malignant etiologies.^4^ Most cases of ALO are caused by tumor recurrence or enteric adhesions. Conventionally, B‐II and R‐Y reconstruction have been thought to preclude endoscopic access because of the acute angle of anastomosis and the long length of the afferent limb.^5^, ^6^, ^7^ Consequently, more invasive procedures, such as percutaneous drainage or open surgery, have been used as the primary treatment for these patients. In recent years, the use of balloon endoscopy has been useful in patients with surgically altered anatomy.^8^, ^9^ Moreover, there has been a remarkable development of endoscopic treatment tools, and there are some reports of balloon‐assisted endoscopic treatment for ALO. In particular, a short double‐balloon endoscopy (DBE) is useful for the endoscopic placement of a self‐expandable metallic stent (SEMS) in malignant ALO. To our knowledge, there are few reported cases of endoscopic treatment for ALO, therefore, the clinical course remains unknown.

In this study, we aimed to evaluate the efficacy and safety of short DBE treatment for ALO.

## MATERIALS AND METHODS

### Study design

This was a single‐center retrospective study approved by the Institutional Review Board of Kansai Medical University (Hirakata, Japan). Using the medical record system, we identified patients who underwent DBE for ALO from January 2009 to December 2020 at Kansai Medical University. We included 22 patients who were diagnosed with ALO and excluded patients with no symptoms or abnormal blood tests or imaging findings. Demographics, surgical variables, post‐endoscopic outcomes, and post‐endoscopic follow‐up details were collected. Patients were divided according to whether the cause was benign or malignant. In this study, we assessed the efficacy and safety of short DBE treatment for ALO.

### Definition of ALO

Patients with ALO usually present with upper abdominal pain, nausea, and vomiting due to the accumulation of secretions in the obstructed jejunum. Acute cholangitis may occur, with patients presenting with fever and typical laboratory findings, such as elevation of bilirubin and aminotransferases. The typical radiographic findings of an obstructed afferent loop consist of a fluid‐filled, dilated afferent limb with fluid that project into the lumen. The diagnosis of ALO was based on the typical symptoms or positive laboratory findings of acute cholangitis as well as the distinct radiographic evidence of afferent loop dilatation.

### Procedure using a short DBE

All DBE procedures were performed by three endoscopists (Masaaki Shimatani, Toshiyuki Mitsuyama, and Norimasa Fukata) with experience in performing 200 or more procedures. and under conscious sedation using intravenous midazolam and/or haloperidol. In each case, the procedure from scope insertion to stent placement was performed by the same endoscopist. Two types of short DBE were used (EI‐530B and EI‐580BT; Fujifilm, Tokyo, Japan), which had a 152‐ and 155‐cm working length with a 2.8‐ and 3.2‐mm working channel, respectively. Since March 2016, we have been using the EI‐580BT at our institution because of the easy SEMS placement with the 3.2‐mm working channel. The soft overtube (TS‐13101 overtube, BS‐2 balloon; Fujifilm) is 105 cm long with an outer diameter of 13.2 mm. All procedures were conducted under carbon dioxide insufflation with a soft transparent hood (DH‐17EN; Fujifilm).

### Definitions and outcome measurement

The following variables were evaluated: technical success rate, clinical success rate, total procedure time, time to reach a target site, recurrent obstruction, time to recurrent obstruction, procedure‐related adverse events, and reintervention. Technical success was defined as successful endoscopic naso‐drainage (END, nasal biliary drainage set, pigtail; Cook Medical, Winston Salem, NC, USA), endoscopic drainage (ED) using a plastic stent (PS; double pigtail; Cook Medical or Medi‐globe, Rohrdorf, Germany) or endoscopic SEMS (Niti‐S; Taewoong Medical, Seoul, South Korea) placement at the intended location. Clinical success was defined as a 50% decrease or normalization of the serum data or improvement of symptoms within 14 days of stent placement. The total procedure time was defined as the interval between scope insertion and removal. The time to reach a target site, obstructive site, or blind end, was defined as the interval from the scope insertion to reaching the afferent limb obstruction. Recurrent obstruction was defined as the composite endpoint of either occlusion or symptomatic migration. The time to recurrent obstruction was defined as the time from SEMS placement to recurrent obstruction for malignant ALO. Stent replacement was performed every 3–6 months for benign ALO, recurrence was not defined. Procedure‐related adverse events were assessed using the American Society for Gastrointestinal Endoscopy severity grading system^10^ and the European Society of Gastrointestinal endoscopy technical review.^11^

**FIGURE 1:**
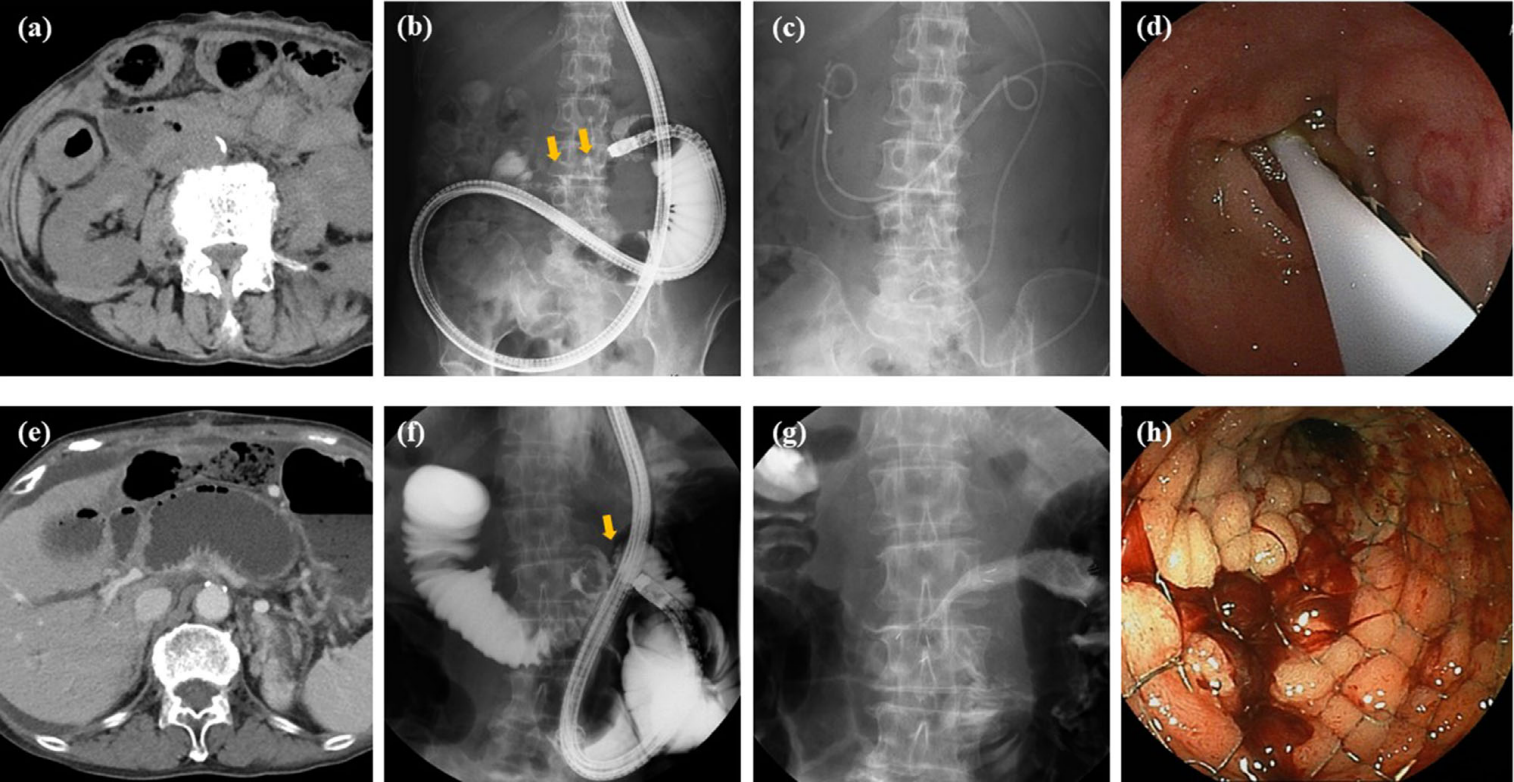
Double‐balloon endoscopic procedure for benign and malignant afferent loop obstruction (ALO). (a) Computed tomography (CT). CT imaging in benign ALO. (b, c) Fluoroscopy, endoscopic naos‐drainage, and endoscopic drainage using plastic stent for benign ALO. Intestinal stenosis (yellow arrow). (d) Endoscopy: endoscopic drainage using plastic stent for benign ALO. (e) CT: CT imaging in malignant ALO. (f, g) Fluoroscopy: endoscopic self‐expandable metal stent (SEMS) placement for malignant ALO. Intestinal stenosis (yellow arrow). (g) Endoscopy: endoscopic SEMS placement for malignant ALO

In cases of benign ALO, END, or ED using a PS was placed across the obstruction site. In the presence of acute cholangitis, bile duct drainage and gastrointestinal obstruction drainage may be required. In contrast, in cases of malignant ALO, SEMS was performed. However, drainage may be performed with END or ED using a PS before SEMS placement for malignant ALO. DBE‐related procedures for benign and malignant ALO are shown in Figure 1.

### Statistical analysis

Continuous variables were expressed as median values. Overall survival (OS) was defined as the time from the diagnosis of ALO to the date of death. In patients still alive at the time of the database lock, censoring was performed at the last follow‐up date (until March 31, 2021). The Kaplan–Meier method was applied to construct curves of time to recurrent obstruction and OS. Statistical analyses were performed using JMP 13.0 (SAS Institute Inc., Cary, NC, USA).

## RESULTS

### Patients

Baseline characteristics of the study population are shown in Table 1. Of the 22 patients with a median age of 70.5 years (interquartile range [IQR] 37–87), 14 (64%) were male. Diseases requiring previous surgery included pancreatic cancer in eight (36%), gastric cancer in seven (32%), hilar cholangiocarcinoma in four (18%), and congenital biliary dilatation in two (9%), and duodenal cancer in one (5%). Reconstruction methods included PD in eight (36%), hepaticojejunostomy with R‐Y in seven (32%), subtotal gastrectomy with R‐Y in four (18%), and total gastrectomy with R‐Y in three (14%). The type of surgery included open surgery in 20 (91%) and laparoscopic surgery in two (9%). In malignant ALO, the cause of ALO was tumor recurrence in 13 (59%). All cases were recurrence with peritoneal dissemination. In benign ALO, the cause of ALO was adhesion in eight (36%) and food impaction in one (5%).

**TABLE 1:** Characteristics of patients with afferent loop obstruction

	**Benign ALO**		**Malignant ALO**		**Total**	
Patients, *n*	9		13		22	
Sex, male, *n* (%)	6	(67)	8	(62)	14	(64)
Age, median (IQR), years	69	(37–79)	71	(51–87)	70.5	(37–87)
Disease requiring the previous surgery, *n* (%)						
Pancreatic cancer	2	(22)	6	(46)	8	(36)
Gastric cancer	2	(22)	5	(38)	7	(32)
Hilar‐cholangiocarcinoma	2	(22)	2	(15)	4	(18)
Congenital biliary dilatation	2	(22)	0	(0)	2	(9)
Duodenal cancer	1	(11)	0	(0)	1	(5)
Type of reconstruction, *n* (%)						
Pancreaticoduodenectomy	2	(22)	6	(46)	8	(36)
Hepaticojejunostomy with R‐Y	4	(44)	3	(23)	7	(32)
Subtotal gastrectomy with R‐Y	3	(33)	1	(8)	4	(18)
Total gastrectomy with R‐Y	0	(0)	3	(23)	3	(14)
Type of surgery, *n* (%)						
Open surgery	7	(78)	13	(100)	20	(91)
Laparoscopic surgery	2	(22)	0	(0)	2	(9)
Main symptom, *n* (%)						
Abdominal pain and/or fever‐up with elevation of hepatobiliary enzymes	5	(56)	7	(54)	12	(55)
Abdominal pain and/or fever‐up without elevation of hepatobiliary enzymes	3	(33)	4	(30)	7	(32)
Jaundice without fever‐up	0	(0)	2	(15)	2	(9)
Vomiting	1	(11)	0	(0)	1	(5)

Abbreviations: ALO, afferent loop obstruction; IQR, interquartile range; R‐Y, Roux‐en‐Y.

### Endoscopic procedure using a short DBE

The outcomes of the endoscopic procedure using a short DBE are shown in Table 2. DBE was able to reach the obstructive site in the afferent limb in all (100%) and reached the blind end in 14 (64%). The technical success rate was 100% (22/22), and the clinical success rate was 95% (21/22). The median time taken to first reach a target site was 17.5 min (IQR 1–93), and the median total procedure time for the first procedure was 61 min (IQR 10–124). Procedure‐related adverse events occurred in two patients (9%). One case was a patient who underwent Kasai surgery and R‐Y reconstruction twice, and a small perforation occurred due to strong adhesions. This patient improved conservative treatment. The other case was a patient who underwent duodenal jejunal anastomosis and R‐Y reconstruction and major perforation occurred due to strong adhesion. This patient underwent surgery. In both cases, the intestinal flexion of the afferent loop was perforated, not the stenotic part. It was thought that the cause was not the scope itself, but the laceration caused by the overtube.

**TABLE 2:** Endoscopic procedure for patients with afferent loop obstruction

	**Benign ALO**		**Malignant ALO**		**Total**	
Patients, *n*	9		13		22	
Technical success, *n* (%)	9	(100)	13	(100)	22	(100)
Clinical success, *n* (%)	8	(89)	13	(100)	21	(95)
Reaching the blind end	7	(78)	7	(54)	14	(64)
Time to first reaching a target site, median, (IQR), min	23	(7–93)	17	(1–57)	17.5	(1–93)
Time to total procedure, median, (IQR), min	62	(10–109)	60	(33–124)	61	(10–124)
Stent placement, *n* (%)						
END	4	(44)	2	(15)	6	(27)
ED using PS	4	(44)	1	(8)	5	(23)
END and ED using PS	1	(11)	0	(0)	1	(5)
Self‐expandable metal stent	0	(0)	10	(77)	10	(45)
Adverse event, *n* (%)	2	(22)	0	(0)	2	(9)

Abbreviations: ALO, afferent loop obstruction; ED, endoscopic drainage; END, endoscopic naso‐drainage; IQR, interquartile range; PS, plastic stent.

In cases of benign ALO, END was performed in four (18%), ED using PS in four (18%), while END and ED using PS in one (5%). In cases of malignant ALO, SEMS was placed in 10 (45%), END was placed in two (9%), and ED using PS was placed in one (5%).

### Recurrent obstruction and reintervention

**FIGURE 2:**
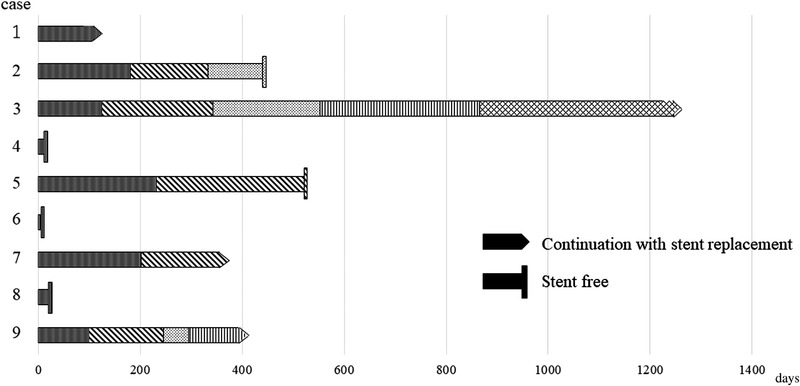
Stent placement in benign afferent loop obstruction. Cases 4, 6, and 8 improved with single stent placement. Cases 2 and 5 improved with the replacement of plastic stent (PS) two and three times respectively. Cases 1, 3, and 9 have been continued to replace PS

The causes of recurrent obstruction are summarized in Table 3. In the case of benign ALO, three of nine (33%) showed improvement of ALO with single stent placement. Two of nine (22%) improved after the replacement of the PS two or three times. Four of nine (44%) continued with the replacement of PS every 3–6 months. Stent placement in benign ALO is shown in Figure 2. In the case of malignant ALO, three of 13 (23%) died following END or ED using PS. Among 10 of the 13 (77%) who underwent SEMS placement, two had stent occlusion prior to death. However, the stent was not replaced in these two patients because their general condition was poor, and the prognosis was considered to be poor. The remaining eight with SEMS did not experience stent occlusion until they died. In brief, reintervention was attempted in six (27%) with benign ALO but was not attempted in malignant ALO.

**TABLE 3:** Long‐term outcome after stent placement

**Benign ALO, *n***	**9**	
Single stent placement, *n* (%)	3	(33)
Replacement of plastic stent two and three times, *n* (%)	2	(22)
Continuously replacement of plastic stent, *n*, (%)	4	(44)

Malignant ALO, *n*	13	
Stent occlusion, *n*, (%)	2	(15)
Stent migration, *n* (%)	0	(0)

Abbreviation: ALO, afferent loop obstruction.

The median follow‐up period was 189.5 days (IQR 7–1527). At the time of final evaluation (March 31, 2021), six (27%, two with benign ALO and four with malignant ALO) were lost to follow‐up, while nine (41%, all patients with malignant ALO) died during follow‐up.

**FIGURE 3:**
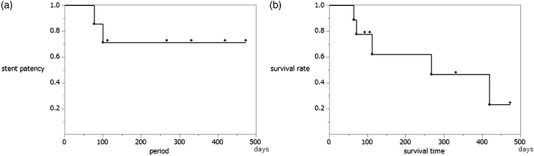
Stent patency and survival time in malignant afferent loop obstruction. (a) Kaplan‐Meier curve of stent patency. (b) Kaplan‐Meier curve of patient survival

The Kaplan‐Meier curve for overall patient survival time is shown in Figure 3a. The median time to patient death was 267 days (IQR 7–473). The Kaplan‐Meier curve for the time to recurrent obstruction is shown in Figure 3b. The median time to recurrent obstruction did not reach the median.

## DISCUSSION

ALO is a rare complication that occurs after gastrointestinal resection. Surgical procedures are the established treatment of choice for ALO.^12^, ^13^, ^14^ However, some patients are poor candidates for a second surgical procedure because of their poor general condition. Bleeding and sepsis have been reported as complications after a PD reoperation.^15^ The percutaneous approach is less invasive than open surgery and has been shown to provide effective palliation;^16^, ^17^, ^18^, ^19^ however, it is difficult to perform percutaneous transhepatic biliary drainage (PTBD) in patients without intrahepatic bile duct dilation or patients with ascites. PTBD includes causes severe adverse events, such as bleeding, biliary peritonitis, acute cholangitis, frequent catheter dislodgement, and poor quality of life. Furthermore, direct puncture of a percutaneous afferent loop is associated with a higher risk of biliary peritonitis.^20^ Therefore, PTBD is an effective treatment method when the endoscopic approach is unsuccessful.

As mentioned above, surgical and percutaneous treatments are effective treatment options for ALO, but they should be avoided in cases of oral administration of antiplatelet agents and anticoagulants as well as cases of ascites accumulation. The endoscopic approach can be used to treat the stenosis more easily and less invasively by using a stent to directly reach the site of obstruction, especially in patients with a high surgical risk.

In this study, 36% of cases failed to reach the blind end, but the intestinal stenosis was improved regardless of not reaching the blind end. As a result, jaundice and cholangitis were controlled even in cases where a bile duct stent was not placed. If these patients were able to be reached the blind end and had cholangitis, biliary stents were placed additionally.

Surgery is the definitive treatment for benign ALO. However, endoscopic intervention can be considered in patients with ALO‐related cholangitis caused by adhesion, enteroliths, and foreign bodies. Both biliary stents and PS placed across the stenosis are often inserted endoscopically, although if the bile duct anastomosis and the intestinal stenosis are close together, a long bile duct stent may be useful when placed on the anal side from the intrahepatic bile duct across the site of stenosis. In our study, 22% of the cases could be treated by only one stent placement, and 33% of the cases were stent‐free after several stent placements. In brief, more than half of the patients were able to maintain a stent‐free status, but the other half had stents replaced every 6 months. Therefore, this procedure may be considered the first step in the treatment of benign ALO, regardless of whether conservative treatment or surgical treatment is needed. Cao et al. reported that 26 patients with benign ALO underwent endoscopically guided insertion of a long nasogastric tube. The nasogastric tube was removed 3–14 days after insertion without recurrent ALO.^21^ However, we experienced cases of immediate recurrence of ALO after endoscopic stent removal. Stent‐free is considered to be a condition in which the PS spontaneously deviates during the indwelling period. There have been no reports of long‐term observations of benign ALO cases that have improved with stent placement. Therefore, this study is a very impressive result. As our balloon‐assisted endoscopic strategy for benign ALO, END, and ED using PS or ED using PS are the first choices in our strategy for benign ALO. This is because it can be judged as healing when the indwelling stent is dislocated spontaneously. In the case of END alone, if recurrence occurs after removal, endoscopic treatment may be required again.

On the other hand, for malignant ALO, the balloon‐assisted endoscopic approach allows for direct identification of the stenosis. If the initial endoscopic treatment reveals malignant findings, SEMS is placed for a period of time. However, if it cannot be determined whether it is malignant or not, a histological evaluation such as a biopsy is performed, and PS is temporarily placed. SEMS is often a candidate treatment for malignant ALO because of its long period of patency. Conventionally, it is not possible to place a SEMS directly because the balloon endoscope has a 2.8‐mm working channel. Therefore, SEMS is placed using a colonoscope or an overtube.^22^, ^23^, ^24^ However, a colonoscope or upper gastrointestinal endoscope can hardly reach the obstruction site in the case of R‐Y reconstruction because of the long intestinal tract. In recent years, a new short DBE with a 3.2‐mm working channel and 152‐cm working length has been used to easily place a SEMS using the through‐the‐scope technique.^25^, ^26^, ^27^, ^28^ Although not applicable in our report, recurrent obstruction after SEMS placement may require re‐intervention in cases with a good prognosis. As a re‐intervention, additional placement of PS or stent‐in‐stent placement of SEMS can be considered. Kida et al. reported that two of 11 malignant ALO patients with successful endoscopic enteral SEMS placement had tumor ingrowth at 9 and 103 days after the first stent was placed, requiring SEMS insertion using the stent‐in‐stent technique.^29^ The previous reports of balloon‐assisted endoscopic treatment for ALO are summarized in Table 4.^19^, ^23^, ^24^, ^25^, ^26^, ^27^, ^28^, ^30^, ^31^, ^32^, ^33^, ^34^, ^35^ Although there are many case reports or case series, there are few comprehensive reports. This study reported nine benign and 13 malignant ALOs.

**TABLE 4:** Balloon‐assisted endoscopic treatment for benign and malignant afferent loop obstruction

	**Author**	**No. patients**	**Primary disease**	**Surgical procedure and reconstruction**	**Endoscopic procedure**	**Endoscopic devise**
Benign	Yane et al.^30^	1	Cholangiocarcinoma	H‐J with R‐Y	END	Standard SBE
	Konishi et al.^31^	1	Biliary Atresia	Kasai portoenterostomy	END	Standard DBE
	Present study	9	Pancreatic cancer 2 Gastric cancer 2 Hilar‐cholangiocarcinoma 2 Congenital biliary dilatation 2 Duodenal cancer	PD 2 H‐J with R‐Y 4 Distal gastrectomy with R‐Y 3	END 4 END and PS PS 4	Short DBE
Malignant	Kida et al.^24^	1	Pancreatic cancer	PD	SEMS (Niti‐s)	Standard DBE
	Sasaki et al.^25^	1	Pancreatic neuroendocrine tumor	PD with R‐Y 1	SEMS (Niti‐s)	Standard DBE
	Fujii et al.^23^	2	Ampullary cancer Cholangiocarcinoma	PD H‐J with R‐Y	SEMS (Niti‐s) SEMS (WallFlex)	Standard DBE
	Nakahara et al.^32^	3	Pancreatic cancer Cholangiocarcinoma Hilar‐cholangiocarcinoma	PD 2 H‐J with R‐Y	SEMS (Niti‐s) 2 END	Standard SBE
	Shugo et al.^33^	1	Pancreatic cancer	PD	SEMS (Niti‐s)	Enteroscope with overtube
	Minaga et al.^27^	1	Duodenal cancer	PD	SEMS (Niti‐s)	Short DBE
	Shimatani et al.^26^	1	Pancreatic cancer 1	PD	SEMS (Niti‐s)	Short DBE
	Tsutsumi et al.^34^	1	Gastric cancer	Total gastrectomy with R‐Y	SEMS (Niti‐s)	Short DBE
	Yane et al.^28^	5	Pancreatic cancer 4 Cholangiocarcinoma	PD 4 Hepatectomy with R‐Y	SEMS (Niti‐s) SEMS (Wall Flex)	Short SBE Standard SBE
	Sasaki et al.^35^	5	Pancreatic cancer 3 Cholangiocarcinoma 2	PD 4 H‐J with R‐Y	SEMS (Niti‐s) 5	Short SBE
	Kanno et al.^19^	1	Gastric cancer	Total gastrectomy with R‐Y	SEMS (Niti‐s)	Short SBE
	Present study	13	Pancreatic cancer 8 Gastric cancer 7 Hilar‐cholangiocarcinoma 4 Congenital biliary dilatation 2 Duodenal cancer	PD 8 H‐J with R‐Y 7 Distal gastrectomy with R‐Y 4 Total gastrectomy with R‐Y 3	SEMS (Niti‐s) 10 END 2 PS	Short DBE

Abbreviations: DBE, double‐balloon endoscopy; END, endoscopic naso‐drainage; H‐J, Hepaticojejunostomy; PD, pancreaticoduodenectomy; PS, plastic stent; SBE, single balloon endoscopy; SEMS, self‐expandable metallic stent; R‐Y, Roux‐en‐Y.

The numbers denote the number of patients that underwent the procedures

In recent years, the endoscopic ultrasound‐guided transgastric approach for the afferent loop using a lumen‐apposing metal stent has been reported in malignant ALO patients presenting with acute cholangitis and failure to reach the afferent loop.^36^, ^37^, ^38^ According to the reports to date, the treatment time is short and there are few complications, and it is considered an alternative treatment to PTBD. Since there is no need to create an external fistula, the patient's quality of life can be maintained. However, endoscopic ultrasound is associated with a risk of peritonitis due to bile leaks. Furthermore, there is a risk of bleeding in patients using oral antiplatelet agents and anticoagulants. Therefore, it is necessary to select a treatment based on the individual patient's condition.

This study has some limitations. First, this was a single‐center retrospective observational study that examined a small number of cases. Since ALO is a rare disease, it is necessary to accumulate cases at multiple centers in order to examine a large sample size. Second, although DBE has become widespread in recent years, it is not available in all institutions. In addition, our facility is specialized because a large number of balloon endoscopic treatments were performed. It is considered that the success rate was very high and there were few complications. Third, in this study, only the results of balloon endoscopic treatment were examined, and it was not a comparative study between PTBD and endoscopic ultrasound. It is necessary to examine these comparative treatments in a larger number of cases in the future.

In conclusion, DBE treatments for ALO were effective and relatively safe because the technical and clinical success rates were very high and there were relatively low complications. Consequently, it can be considered the first‐line treatment for both benign and malignant ALO.

## CONFLICT OF INTEREST

The authors declare no conflict of interest.

## FUNDING INFORMATION

This study has received no financial support.
